# Isotretinoin and Suicide: Data Mining of the United States Food and Drug Administration Adverse Event Reporting System Database

**DOI:** 10.7759/cureus.70502

**Published:** 2024-09-30

**Authors:** Ravindra K Ganjikunta, Rupeshkumar B Naik, Chandrasekhar Vallepalli, Jegadeeshkrishnan M

**Affiliations:** 1 Pharmacology, Sri Venkateswara Institute of Medical Sciences, Tirupati, IND; 2 Forensic Medicine, Sri Venkateswara Institute of Medical Sciences, Tirupati, IND; 3 Community Medicine, Sri Venkateswara Institute of Medical Sciences, Tirupati, IND

**Keywords:** acne, adolescents, adverse events, chi-square test, faers, isotretinoin, suicide

## Abstract

Background

Isotretinoin is a 13-cis-retinoic acid most commonly used to treat severe nodular acne. It may result in psychiatric adverse events, such as completed suicide, even though the exact mechanism is unknown. Despite extensive research, the potential link between isotretinoin and psychiatric side effects remains unclear.

Objectives

The main objective of this study is to find the relationship between isotretinoin and suicide-related adverse events by analyzing the United States Food and Drug Administration Adverse Event Reporting System (FAERS).

Methods

The study data were obtained from the FAERS database from 1982 to March 2024. The inclusion criteria were: (i) isotretinoin-related suicide and suicide-related adverse events; (ii) reports with specified age and gender. Out of the 73,076 extracted cases, 2839 were finalized for analysis based on the inclusion criteria. The data were represented as frequency and percentages. A chi-square or Fischer exact test was used to analyze the categorical variables. The P-value <0.05 was considered as statistically significant.

Results

As 2433 patients experienced multiple reactions, the reported suicide and suicide-related adverse events totalled 3,059. The most commonly affected individuals were males, with the exception of depression suicidal event, which was more common in females. Among all age groups, those aged 10-19 were the most affected, as the occurrence of acne and the use of isotretinoin were higher in this age group. Almost all the reactions were serious, and there were 349 completed suicides.

Conclusion

The usage of isotretinoin can increase the possibility of suicide-related adverse events and should be used cautiously, especially in adolescents and patients with previous psychiatric disorders.

## Introduction

Isotretinoin, a synthetic vitamin A derivative, has been widely used to treat severe recalcitrant nodular acne since its approval by the U.S. Food and Drug Administration (FDA) in 1982 [1]. Although the success rate in treating recalcitrant acne is well-established, concerns have been raised about a possible link to psychological side effects, such as anxiety, depression, suicidal thoughts, and attempts [2].

Spontaneous reporting of adverse drug events suggested a correlation between isotretinoin and suicide-related adverse events. However, isotretinoin can cause other side effects such as alopecia, hypertriglyceridemia, teratogenic effects, etc. Importance arose for suicidal adverse events as the frequency of occurrence increased over time. In the year 2019, there were 12 reported deaths in the United Kingdom among individuals who had been prescribed isotretinoin, with 10 of these deaths resulting from suicide [3]. The causal association between isotretinoin use and suicide risk remains unclear, with conflicting results in different studies [4,5]. There is uncertainty about the exact mechanism by which isotretinoin may worsen psychological side effects. However, some concepts have been proposed to explain the effects of drugs on the nervous system, cellular metabolism, and inflammatory processes [6]. Hence, to investigate this relationship, some studies have utilized this FDA Adverse Event Reporting System (FAERS) database [2,7]. The FAERS database is a spontaneous reporting system that contains information about adverse events associated with marketed drugs [8].

Our aim in this study is to analyze FAERS data to explore the potential association between isotretinoin and suicide-related adverse events by assessing demographic parameters like age and gender, as well as outcome and categorization of the reactions.

We intend to deliver the safety profile of isotretinoin and information about future risk mitigation strategies in this article.

## Materials and methods

Data source

The FAERS database includes adverse event reports, medication error reports, and product quality complaints. These reports are submitted by healthcare professionals (HCPs), consumers, manufacturers, patients, and other reporters to the FDA. This database is updated once every three months, i.e., quarterly every year (Q1-Q4) [8].

In the FAERS Public Dashboard, the isotretinoin product was entered into the search field, resulting in a listing of cases that were subsequently opened and downloaded (yyyy and q present the year and quarter, respectively) in the .xlsx Excel file (Line Listing(dxjZy)) (Microsoft® Corp., Redmond, WA) format from 1982Q1 to 2024Q1 on May 6, 2024. This file contains all adverse drug events associated with isotretinoin that have been reported to the FDA. It has 24 columns; among those, we considered patient demographics, drug information, reactions, and outcomes columns as variables for our analysis.

The institutional review board approval is not required for this study, as FAERS data are publicly available.

Study design

The inclusion criteria were: (i) isotretinoin-related suicide and suicide-related adverse events from the FAERS database, and (ii) reports with specified age and gender were included in our study. Based on Medical Dictionary for Regulatory Activities version 27.0 (MedDRA), we have included all drug-associated suicide-related preferred terms (PT) such as completed suicide, depression suicidal, suicidal behaviour, suicidal ideation, suicide attempt, and suicide threat for our analysis.

The exclusion criteria were adverse events other than suicide and suicide-related events. These data contained 26 duplicate cases after applying inclusion and exclusion criteria. The final analysis was done with 2839 cases.

Statistical analysis

The data were entered into Microsoft Excel 2021. Descriptive statistical analysis was performed, and categorical variables were represented as frequencies and percentages. For categorical variables, the chi‑square or Fisher’s exact test was used. A P-value < 0.05 was considered statistically significant. Statistical analysis was done using the SPSS version 26.0 statistical software (SPSS Inc., Chicago, IL, USA).

## Results

**Figure 1:**
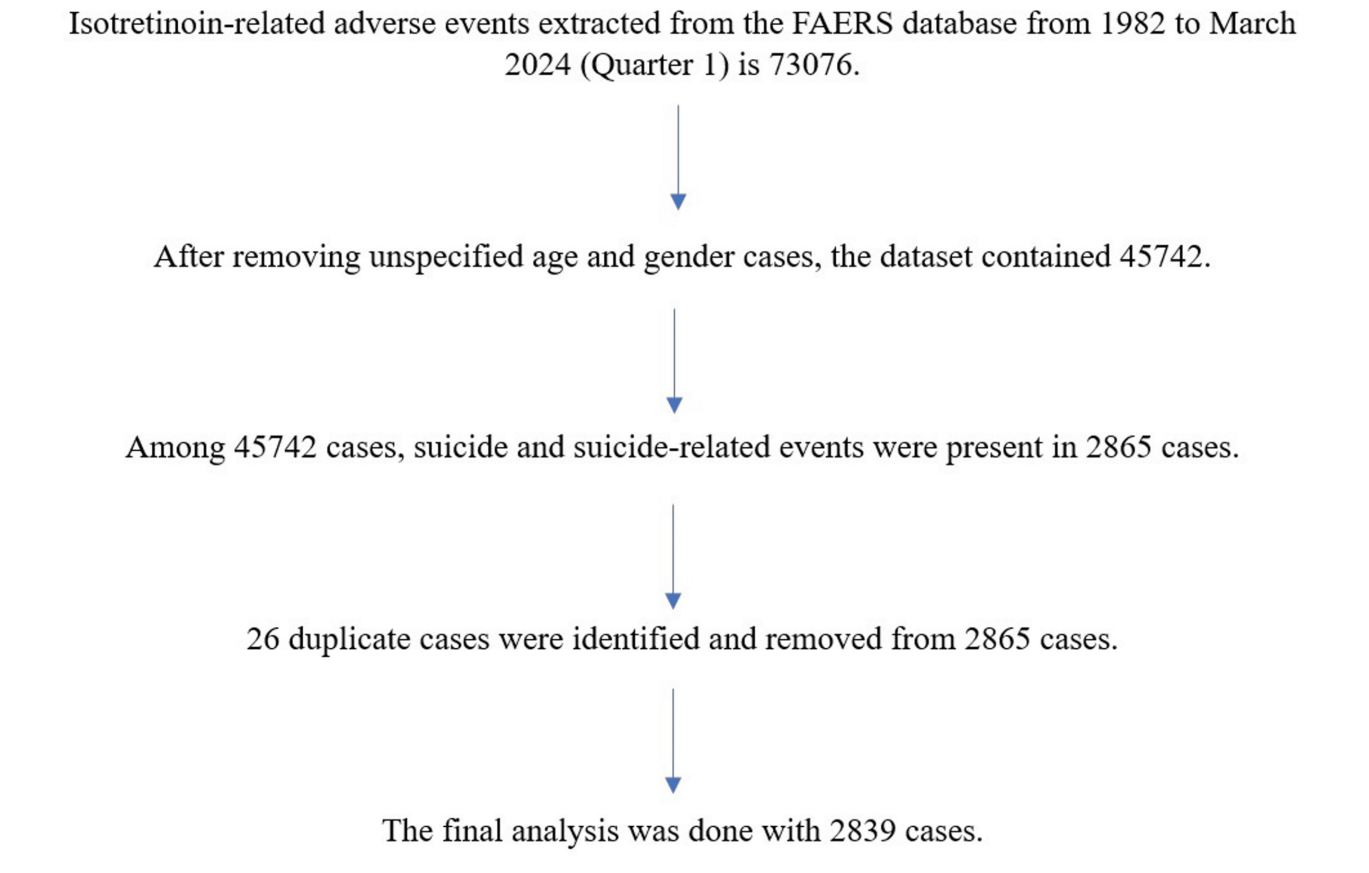
Flow chart of data extraction process from FAERS database FAERS: FDA Adverse Event Reporting System

A total of 73,076 isotretinoin-induced adverse event reports were submitted to FAERS from 1982 to 2024 Q1 (March 2024). Age and gender were not specified in 27,334 reports and they were removed. Among the 45,742 reports, suicide and suicide-related adverse events reports were 2,865. After removing the duplicates of 26 cases, the final reports to analyze were 2,839. Out of these 2839 cases, 66 cases were literature reports, and 2773 were spontaneous reports. The data extraction process of isotretinoin-related suicide and suicide-related events done from the FAERS database is explained in Figure 1.

The age groups we have considered were 0-9, 10-19, 20-29, 30-39, 40-49 and ≥50. In our analysis, the mean age and standard deviation (mean age ± SD) were 21.22 ± 8.39. The 0-9 year-age group accounted for one patient (0.04%), the 10-19 year-age group accounted for 1694 patients (59.67%), the 20-29 year-age group accounted for 744 patients (26.21%), the 30-39 year-age group accounted for 263 patients (9.26%), the 40-49 year-age group accounted for 106 patients (3.73%), and ≥50 year-age group accounted for 31 patients (1.09%). The number of patients presented in each age group was mentioned in Table 1.

**Table 1:** Age group-wise distribution of patients

S. No	Age group (years)	Number of patients (%)
1.	0–9	1 (0.04)
2.	10–19	1694 (59.67)
3.	20–29	744 (26.21)
4.	30–39	263 (9.26)
5.	40-49	106 (3.73)
6.	≥50	31 (1.09)
	Total	2839 (100)

Of 2,839 cases, 56.92% were males and the remaining 43.08% were females. The total number of reported suicide and suicide-related adverse events was 3,059, as 2,433 patients experienced multiple events, that is, two or more. Among the 3059 events, suicidal ideation (63.98%) was reported as the highest event, followed by suicide attempt (22.56%), completed suicide (11.41%), depression suicidal (1.21%), suicidal behaviour (0.75%), and suicide threat (0.09%). A statistically significant difference (p<0.05) was observed for completed suicide and suicidal ideation events with respect to gender. The association of gender with suicide-related adverse events is mentioned in Table 2.

**Table 2:** Association of gender with suicide-related adverse events *Reactions are categorized based on MedDRA Preferred terms (PT)

S. No	Adverse event (PT)^*^	Males (%)	Females (%)	Total number of adverse events (%)	Chi-square	P-value
1.	Completed suicide	274 (78.5)	75 (21.5)	349 (11.41)	75.631	<0.001
2.	Depression suicidal	18 (48.6)	19 (51.4)	37 (1.21)	1.046	0.320
3.	Suicidal behaviour	13 (56.5)	10 (43.5)	23 (0.75)	0.002	1.000
4.	Suicidal ideation	1073 (54.8)	884 (45.2)	1957 (63.98)	11.25	0.001
5.	Suicide attempt	371 (53.8)	319 (46.2)	690 (22.56)	3.696	0.057
6.	Suicide threat	3 (100)	0 (0)	3 (0.09)	2.273	0.26
		Total number of adverse events		3059		

Among the 3059 reported adverse events, the majority (59.37%) occurred in the 10-19 age group. The occurrence of suicide and suicide-related events in other age groups was 26.12% in the 20-29 age group, 9.61% in the 30-39 age group, 3.76% in the 40-49 age group, 1.11% in the ≥ 50 age group, and 0.03% in the 0-9 age group. The statistically significant (p < 0.05) differences among the age groups with adverse events were found in completed suicide, suicidal behaviour, suicidal ideation, and suicidal attempt. The suicide and suicide-related adverse events accounted for specific age groups were mentioned in Table 3.

**Table 3:** Association of age groups with suicide-related adverse events

S. No	Adverse event	Age group (years)							Chi-square	P-value
		0–9 (%)	10–19 (%)	20–29 (%)	30–39 (%)	40–49 (%)	≥50 (%)	Total number of adverse events (%)		
1.	Completed suicide	0 (0)	202 (57.88)	120 (34.38)	22 (6.3)	4 (1.15)	1 (0.29)	349 (11.4)	23.77	<0.001
2.	Depression suicidal	0 (0)	20 (54.05)	8 (21.62)	6 (16.22)	1 (2.70)	2 (5.41)	37 (1.21)	8.96	0.111
3.	Suicidal behaviour	0 (0)	7 (30.43)	6 (26.09)	9 (39.13)	1 (4.35)	0 (0)	23 (0.75)	25.93	<0.001
4.	Suicidal ideation	1 (0.05)	1127 (57.59)	518 (26.47)	198 (10.12)	89 (4.55)	24 (1.23)	1957 (63.98)	22.37	<0.001
5.	Suicide attempt	0 (0)	459 (66.52)	146 (21.16)	58 (8.41)	20 (2.90)	7 (1.01)	690 (22.56)	18.83	0.002
6.	Suicide threat	0 (0)	1 (33.33)	1 (33.33)	1 (33.33)	0 (0)	0 (0)	3 (0.09)	2.43	0.787
	Total number of adverse events (%)	1 (0.03)	1816 (59.37)	799 (26.12)	294 (9.61)	115 (3.76)	34 (1.11)	3059 (100)		

## Discussion

We analyzed data on isotretinoin-induced suicide and suicide-related adverse events downloaded from the FAERS database from the year 1982 to March 2024 (Q1). The total number of cases analyzed was 2839. However, 2433 patients experienced multiple adverse events, bringing the total number of adverse events to 3059.

The mean age and standard deviation (mean age ± SD) of our study were 21.22 ± 8.39. However, a prospective study conducted in the Czech Republic shows that the mean age of patients was 18.1 years [9]. In our study, the 10-19 age group consists of the majority (59.67%) of patients, so the majority (59.37%) of isotretinoin-related adverse events also occurred in the same age group. Acne affects 85-90% of adolescents. Of all types of acne, moderate to severe forms account for 15 to 20% [10-12]. Also, here 99.6% of the reports were serious.

It is also important to recognize other psychiatric events such as attention deficit hyperactivity disorder (ADHD), anxiety disorders, bipolar and mood disorders, eating disorders, emotional lability, insomnia, psychotic disorders, and psychotic symptoms. However, we have analyzed suicide and suicide-related events. These are more serious in nature, and a highly important group needs to be focused on.

Isotretinoin is the only non-psychotropic drug in the top 10 list of drugs most commonly reported to be associated with depression [1]. It is also known as 13-cis-retinoic acid. Similar to vitamin A, it belongs to the retinoid class of drugs [13]. Two studies conducted on mice and rats showed an increase in depressive behaviour as a result [14,15]. The proposed mechanism in animals is that it affects the trophic processes in the brain and may reduce the volume of the hippocampus [16].

In humans, depression and suicide-related events usually occur one to two months after the initiation of treatment with it. The suggested mechanism that causes adverse mood-related events is the effect of retinoic acid on either the alteration of neuroplasticity or the metabolic process. This can also be explained by altering the levels of neurotransmitters involved in mood regulation, such as dopamine, serotonin, and norepinephrine [1,17,18]. It is not due to the immediate alterations in neurotransmitters, like antidepressants; here also, the reflection in behavioural changes will also take months [19].

Among the 3059 events, suicide-related adverse events occurred more frequently in males than in females, with the exception of depression suicidal event. This event occurrence was slightly higher in females (51.4%) than males (48.6%). Generally, females are more prone to emotional distress, which increases the chance of suicidal ideation. However, in our study, we found a statistically significant (p < 0.05) difference between genders regarding the adverse events of completed suicide and suicidal ideation, which were reported more in males. The social stigma surrounding mental health may lead to underreporting from females, which is the probable explanation here.

Similar to our study, another study by Singer et al. stated that suicide-related adverse events occurred more frequently in males [7]. Although this study was also based on the FAERS database, our study covers the maximum time period, which is from 1982 to March 2024. However, studies conducted by Lappas et al. and Droitcourt et al. showed that these results were contradictory to our study [4,20].

Among the 3059 suicide-related events in our analysis, the 10-19 age group is more affected, and the majority (59.37%) of the events occurred in this age group, followed by the 20-29 age group (26.12%), as the occurrence of acne and the usage of isotretinoin are more common in these age groups. Based on MedDRA, suicide-related adverse events were classified into six categories. Except for suicidal behaviour (39.13%), which was more common in the 30-39 age group, all the rest were highest in the 10-19 age group only. As the adolescent period is highly vulnerable to emotional and psychological alterations due to hormonal fluctuations and social pressures, this age group is at increased risk of suicide-related events. Isotretinoin use may inadvertently exacerbate underlying psychological vulnerabilities, leading to a higher frequency of suicide-related adverse events in this group compared to others.

In this study, among the six categories of adverse events, suicidal ideation occurred the highest (63.98%), and the lowest was suicide threat (0.03%). A similar study done by Singer et al. also reported that suicidal ideation was the highest (12.8%) among other suicide-related adverse events, which is similar to our study results [7]. A study conducted in Europe reported that mood changes were the highest in 6.66% of patients, and suicidal ideation occurred in 0.02%. However, this prospective study monitored adverse events related to all systems [21].

In accordance with our study, where males (78.5%) were highest among all completed suicide cases, studies done by Wysowski et al. and Droitcourt et al. also reported that the occurrence of completed suicides was highest in males with 84% and 82.4%, respectively [2,20]. Patients with family history and past history of psychiatric illness could also have contributed to the completed suicide event as confounders.

Limitations

These reports were predominantly from the United States and the United Kingdom, with a few from other regions. The incidence of adverse events cannot be calculated using the FAERS database because it is retrospective data. It contains spontaneous reporting, which precludes comprehensive assessment due to limited information, underreporting, and selective reporting, which might have caused bias. Additionally, some cases involved multiple suspect drugs, including SSRIs (selective serotonin reuptake inhibitors), atypical antidepressants, and atypical antipsychotics, which may also have contributed to the events. Although depression events are important to consider here, we focused on depression suicidal events instead of simple depression, which is more relevant to suicidal-related adverse events.

## Conclusions

Based on our study data, isotretinoin may increase the risk of suicide and suicide-related adverse events. The adolescent age group was affected more often than other age groups. Also, males were affected more often than females were here. However, some studies suggest that suicide-related adverse events are more common in females. The exact mechanism involved in causing suicide and suicide-related adverse events due to isotretinoin remains to be identified. According to our study, isotretinoin should be used cautiously in adolescent patients, and the suicidal risk should be conveyed to the patients and patient caregivers. Patients with a family history of psychiatric illness are more likely to be affected by suicidal thoughts, so prescribing isotretinoin to these patients can be avoided.

## References

[1] Bremner JD, McCaffery P (2008). The neurobiology of retinoic acid in affective disorders. Prog Neuropsychopharmacol Biol Psychiatry.

[2] Wysowski DK, Pitts M, Beitz J (2001). An analysis of reports of depression and suicide in patients treated with isotretinoin. J Am Acad Dermatol.

[3] Mahase E (2019). Isotretinoin: experts convene to investigate new concerns over suicide risk. BMJ.

[4] Lappas AS, Edwards Suarez L, Tzanetakou V, Morton S, Schofield C, Christodoulou NG (2022). Factors associated with increased suicidality risk following referral for isotretinoin commencement. Australas Psychiatry.

[5] Hersom K, Neary MP, Levaux HP, Klaskala W, Strauss JS (2003). Isotretinoin and antidepressant pharmacotherapy: a prescription sequence symmetry analysis. J Am Acad Dermatol.

[6] Karadag AS, Ertugrul DT, Takci Z, Bilgili SG, Namuslu M, Ata N, Sekeroglu R (2015). The effect of isotretinoin on retinol-binding protein 4, leptin, adiponectin and insulin resistance in acne vulgaris patients. Dermatology.

[7] Singer S, Tkachenko E, Sharma P, Barbieri JS, Mostaghimi A (2019). Psychiatric adverse events in patients taking isotretinoin as reported in a Food and Drug Administration database from 1997 to 2017. JAMA Dermatol.

[8] (2024). FDA adverse event reporting system (FAERS) (Internet). https://www.fda.gov/drugs/drug-approvals-and-databases/fda-adverse-event-reporting-system-faers.

[9] Nevoralová Z, Dvořáková D (2013). Mood changes, depression and suicide risk during isotretinoin treatment: a prospective study. Int J Dermatol.

[10] White GM (1998). Recent findings in the epidemiologic evidence, classification, and subtypes of acne vulgaris. J Am Acad Dermatol.

[11] Yahya H (2009). Acne vulgaris in Nigerian adolescents--prevalence, severity, beliefs, perceptions, and practices. Int J Dermatol.

[12] Dawson AL, Dellavalle RP (2013). Acne vulgaris. BMJ.

[13] Bremner JD, Shearer KD, McCaffery PJ (2012). Retinoic acid and affective disorders: the evidence for an association. J Clin Psychiatry.

[14] O'Reilly KC, Shumake J, Gonzalez-Lima F, Lane MA, Bailey SJ (2006). Chronic administration of 13-cis-retinoic acid increases depression-related behavior in mice. Neuropsychopharmacology.

[15] Trent S, Drew CJ, Mitchell PJ, Bailey SJ (2009). Chronic treatment with 13-cis-retinoic acid changes aggressive behaviours in the resident-intruder paradigm in rats. Eur Neuropsychopharmacol.

[16] O'Reilly K, Bailey SJ, Lane MA (2008). Retinoid-mediated regulation of mood: possible cellular mechanisms. Exp Biol Med (Maywood).

[17] Charest A, Wainer BH, Albert PR (1993). Cloning and differentiation-induced expression of a murine serotonin1A receptor in a septal cell line. J Neurosci.

[18] Matsuoka I, Kumagai M, Kurihara K (1997). Differential and coordinated regulation of expression of norepinephrine transporter in catecholaminergic cells in culture. Brain Res.

[19] Oliveira JM, Sobreira G, Velosa J, Telles Correia D, Filipe P (2018). Association of isotretinoin with depression and suicide: a review of current literature. J Cutan Med Surg.

[20] Droitcourt C, Poizeau F, Kerbrat S (2020). Isotretinoin and risk factors for suicide attempt: a population-based comprehensive case series and nested case-control study using 2010-2014 French Health Insurance Data. J Eur Acad Dermatol Venereol.

[21] Brzezinski P, Borowska K, Chiriac A, Smigielski J (2017). Adverse effects of isotretinoin: a large, retrospective review. Dermatol Ther.

